# Chronology of prescribing error during the hospital stay and prediction of pharmacist's alerts overriding: a prospective analysis

**DOI:** 10.1186/1472-6963-10-13

**Published:** 2010-01-12

**Authors:** Thibaut Caruba, Isabelle Colombet, Florence Gillaizeau, Vanida Bruni, Virginie Korb, Patrice Prognon, Dominique Bégué, Pierre Durieux, Brigitte Sabatier

**Affiliations:** 1 Department of pharmacy, APHP, Georges Pompidou European Hospital, 75015 Paris, France; 2 Laboratoire Interdisciplinaire de Recherche en Economie de Santé, Paris, France; 3 Department of Hospital Informatics, Evaluation and Public Health, APHP, Georges Pompidou European Hospital, 75015 Paris, France; 4 INSERM, UMR S 872, Equipe 20, Paris, France; 5 Centre de Recherche des Cordeliers, Université Paris Descartes, Paris, France; 6 INSERM, Centre D'investigation Épidémiologique 4, Paris, France; 7 Université Paris Descartes, INSERM U765, 4 avenue de l'Observatoire, 75006 Paris, France

## Abstract

**Background:**

Drug prescribing errors are frequent in the hospital setting and pharmacists play an important role in detection of these errors. The objectives of this study are (1) to describe the drug prescribing errors rate during the patient's stay, (2) to find which characteristics for a prescribing error are the most predictive of their reproduction the next day despite pharmacist's alert (*i.e*. override the alert).

**Methods:**

We prospectively collected all medication order lines and prescribing errors during 18 days in 7 medical wards' using computerized physician order entry. We described and modelled the errors rate according to the chronology of hospital stay. We performed a classification and regression tree analysis to find which characteristics of alerts were predictive of their overriding (*i.e*. prescribing error repeated).

**Results:**

12 533 order lines were reviewed, 117 errors (errors rate 0.9%) were observed and 51% of these errors occurred on the first day of the hospital stay. The risk of a prescribing error decreased over time. 52% of the alerts were overridden (*i.e *error uncorrected by prescribers on the following day. Drug omissions were the most frequently taken into account by prescribers. The classification and regression tree analysis showed that overriding pharmacist's alerts is first related to the ward of the prescriber and then to either Anatomical Therapeutic Chemical class of the drug or the type of error.

**Conclusions:**

Since 51% of prescribing errors occurred on the first day of stay, pharmacist should concentrate his analysis of drug prescriptions on this day. The difference of overriding behavior between wards and according drug Anatomical Therapeutic Chemical class or type of error could also guide the validation tasks and programming of electronic alerts.

## Background

Drug prescribing errors are defined as a prescribing decision or prescription writing process that results in an unintentional, significant reduction in the probability of treatment being timely and effective or increase in the risk of harm, when compared with generally accepted practice. High rates of inpatient prescribing errors have been reported: 1.5-5.3 per 100 drug orders, or 1.4 errors per admission [1,2].

In the context of inpatient care, both Computerized Physician Order Entry (CPOE) implemented with Clinical Decision Support Systems [3-7] and the review of drug orders by pharmacists (hereafter referred to as 'pharmacy validation') [8-10], can reduce the rate of errors. The prevention of prescribing errors implies that the physician captures his/her prescriptions in the CPOE and a pharmacist analyses them the same day to detect a prescribing error. In case of error, the pharmacist notifies the physician by phone or through electronic alert in the patient record. The physician can modify the prescription, complying or not to the alert. We showed in a previous study that prescribers override 70% of pharmacists' alerts [11].

In the hospital setting, some studies suggest that prescribing errors could preferably occur at hospital admission, during a transfer or at discharge [12,13]. To our knowledge, no study described the daily distribution of drug prescribing errors over the hospital stay.

The first objective of this study is to describe the rate of prescribing errors on first day of patient's stay and on the 14 following days. The second objective is to find which characteristics for an alert are the most predictive of its overriding.

## Methods

### Setting

In France, the physician is entirely responsible for prescriptions, including specification of the brand name of the drug (rather than its international denomination), infusion time and solution for reconstitution of intravenous medication. In this context, a pharmacist must alert the prescriber in cases of unavailability or non-conformity with best practice. However, the prescriber cannot modify the prescription directly, with the exception of replacing one drug with another having the same international denomination. Nurses should administer exactly what is written on the prescribing order.

Georges Pompidou European Hospital (HEGP) is a French tertiary care university hospital with 717 beds. A patient information system, integrating an electronic patient record and a CPOE (Dx-Care^®^, Medasys™) is implemented throughout the hospital since its inception in 2000. Dx-Care^® ^is at the centre of care delivery. It is used by doctors, pharmacists and nurses:

• to prescribe laboratory examinations and imaging tests for a patient,

• to visualize the results of laboratory tests,

• to establish and to consult nursing schedules,

• to archive a structured observation,

• to prescribe drugs,

• to validate prescriptions by pharmacists (pharmacy validation).

The drug prescription facility is available in 17 medical wards, 506 beds, 70% of the hospital's beds. The remaining wards, which do not use the CPOE are the ones for oncology (15% of hospital beds) and for emergency or intensive care (15%). Pharmacy validation is carried out daily, from Monday till Friday, in 7 wards (148 beds) out of the 17 which use the CPOE: immunology, nephrology, vascular medicine, geriatrics, diabetes care and internal medicine (2 wards). It is performed twice a week in 7 other wards (300 beds), and the drug orders of the 3 remaining wards (58 beds) are not reviewed at all by any pharmacist.

Five full-time pharmacists are involved in pharmacy validation. Nights' prescriptions are reviewed on the next day and weekends' prescriptions are reviewed on Monday if unexpired.

We define a prescription as a list of drug orders made per day by physician for one patient. For each drug order the physician has to precise: drug name, dose, unit, reconstitution process, route and optional annotation in a plain text field. In addition, the physician has to choose frequency and duration of the order. Various types of prescription aid are available: information about reconstitution processes for intravenous drugs, typical orders pre-specified by pharmacists for intravenous drugs and an integrated drug-drug interaction system are to be targeted by the prescriber. Only alert concerning maximum dose for oral drugs are actively targeted by the system without request by the prescriber.

The pharmacist analyses each patient's prescription, drug order by drug order (dose, unit, time to take, route, frequency in a day, reconstitution process) and the prescription as a whole, testing for drug-drug interactions. The pharmacist has access to biological data and patient record. This analysis is performed daily so that a drug order prescribed for 3 days is validated 3 times.

In case of prescribing error, the pharmacist posts a message, which can be visualized by prescribers through an 'accepted' or 'refused' symbol inserted next to the order line. The 'accepted' symbol indicates that the pharmacist agrees with the prescription, unless a comment is added relating to good practice, which may or may not suggest a modification of the prescription line. The 'refused' symbol indicates that the pharmacist disagrees with the prescription line, having identified a potential severe prescribing error and suggesting its correction. The physician may click on the symbol to visualize the pharmacist's comment, but is not obliged to take that comment into account. We define as alert, any line with a 'refused' symbol or an 'accepted' symbol associated with a comment from the pharmacist and which corresponds to a potential prescribing error. The prescriber can choose to ignore the alert and maintain his/her order along with the pharmacist symbol: in this case we considered the alert as overridden. Alternatively, the prescriber may take the alert into account. Then, the prescriber can either discontinue the order or modify it by cancelling the order line and recreating another one. It is therefore possible from the pharmacy validation database to analyse whether or not an alerts has been overridden the following day.

### Collection of data

The 7 participating wards are those where pharmacy validation is performed daily. Prescribers of these wards were 24 physicians. All data were collected prospectively by pharmacists while validating the prescriptions. According to French regulation, this study that aimed at improving quality of care did not require to be approved by any research ethic committee. The data collection has been approved by French Data Protection Authority. Pharmacists collected prescribing errors detected for each patient admitted in these 7 wards between June 26 to July 13, 2007. We defined as "*new prescribing error*" any error appearing for the first time in the patient's prescription whether at admission or in the following days. If this error was maintained the next day, it was not any more considered as "new". Only new errors were recorded and for each of them, their chronology of appearance during the patient's stay (*i*^*th *^day), their type and degree of severity, as defined according to a commonly used classification (see Additional file 1) [3,6,8,14,15]. For each patient admitted during the study period we also collected data regarding renal impairment, hypertension, thromboembolic disease, as these are clinical contexts known to be associated with more prescribing errors [16,17].

Since pharmacy validation is provided from Monday to Friday, any prescribing error occurring during the week-end was only collected if still present on following Monday. Thanks to the Dx-Care^® ^program we still had access to the exact date of this error, therefore to its chronology in hospital stay. Our first objective consisted in describing the chronology of all new prescribing errors.

Our secondary objective relates to alerts posted by pharmacists in response to new prescribing errors. The alert was considered as overridden if repeated on following days for the same order, consecutively to the absence of any modification of prescription neither evolution of clinical context.

### Statistical analysis

#### Primary outcome

We first described the rate of new prescribing error according to its chronology in the patient's hospital stay. The day of admission was noted as day one.

Then we modelled the time-errors relationship in the first seven days of stay with multivariable models (we ruled-out prescriptions recorded after the seven first days of stay as the number of new errors decreased significantly after this day inducing estimation problems). We tested patient medical data (renal impairment, hypertension and thromboembolic disease), wards, number of order lines, day of discharge as potential confounders of the model. Renal impairment was defined according to the estimated glomerular filtration rate (less than 79 mL/min/1.73 m^2^). Hypertension was defined according to the arterial blood pressure (systolic blood pressure of 140 mm Hg or greater or a diastolic blood pressure of 90 mm Hg or greater). Thromboembolic disease was defined by the prescription of an anticoagulant drugs.

We modelled the risk of an error on *i*^*th *^day of stay with a Generalized Estimating Equation (GEE) regression model taking account for dependency between repeated measurements in a stay. In addition, a mixed Poisson regression model was performed to examine whether the number of new prescribing errors per order lines was related to *i*^*th *^day of stay. We presented the results from the Poisson regression model as estimation of the number of new prescribing errors per 10 order lines since the median number of order lines in a prescription was 7. For both models, a backward selection process with all potential confounders was used to reach the final multivariate model, and the *i*^*th *^day of stay was introduced as a discrete or a continuous variable with possibly mathematical transformation according to the minimum deviance criteria.

#### Secondary outcome

To find which characteristics of new alerts were the most predictive of alert's overriding, we performed a classification and regression tree (CART) analysis. This method developed by Breiman et al. [18] consisted in algorithms with logical "if-then" conditions for predicting or classifying cases. We chose this method since it produced simple decision rules allowing pharmacists to classify prescribing errors with low or high risk of alert's overriding.

We removed new alerts occurring on the day of discharge or after the fifteenth day of stay because it was not possible to allocate them a status (overridden the next day or not). Additionally, weekends' prescriptions were not present in our sample since the pharmacist didn't review prescriptions on these days. The potential variables of interest included in the model were medical history (renal impairment, hypertension, thromboembolic disease), wards, Anatomical Therapeutic Chemical classification drug, number of order lines, type of alerted error, severity of alerted error and the *i*^*th *^day in the stay. The GINI criterion was used to determine the best split at each node. The tree was pruned according to the one standard error rule with a 50-fold cross validation procedure [18]. Since we aimed to maximize the number of true positive alerts (i.e. alerts predicted as overridden which would actually be) and to minimize the number false positive alerts (i.e. alerts not overridden the next day which are predicted as overridden), we respectively maximised positive predictive value and specificity introducing weighing factors of misclassification.

All statistical analyses were performed using SAS 9.1 (SAS Institute, Cary, North Carolina, United States) and R software (version 2.7.2).

## Results

### Participants

A total of 204 patients with 214 stays of at least 24 hours (1 594 hospital days) were included in the study: 198 patients (97.1%) had one stay, 6 (3.0%) two stays or more. Median length of stay for the patient was 6 days (inter quartile range from 3 to 11 days). Mean number of order lines in a prescription was 7.9 ± 4.2. Thirty six percent stays took place in both internal medicine wards, 17% in clinical immunology, 15% in vascular medicine, 12% in geriatrics, 11% in diabetes care and 9% in nephrology.

Eighty four patients (41%) had none of these 3 comorbidities: renal impairment, hypertension and thromboembolic disease. Eighty seven (43%) patients had only one co-morbidity, twenty eight patients (14%) had 2 co-morbidities and 5 patients (2%) had 3 co-morbidities. Hypertension, renal impairment and thromboembolic disease concerned respectively 90 patients (44%), 56 patients (27%) and 32 patients (16%).

### Outcomes

#### Primary outcome

During the study period, pharmacists reviewed 12 533 order lines for the 204 patients admitted in the 7 wards (*i.e*. 214 stays). Pharmacist detected 117 new prescribing errors (*i.e*. prescribing error detecting for the first time) and these new prescribing errors concerned 77 stays (36% of the stays). Among these errors, 103 occurred Monday through Friday and 14 between Saturday and Sunday.

**Figure 1:**
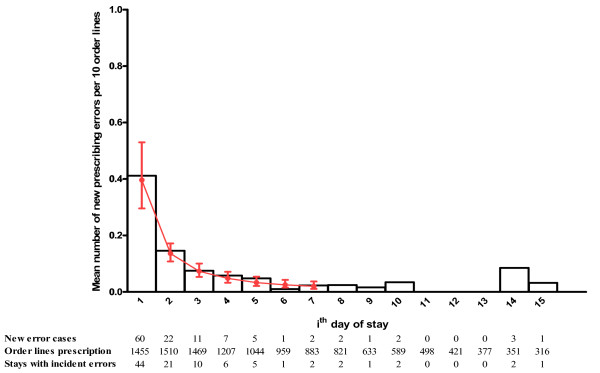
**Number of new prescribing errors per 10 order lines by *i*^th ^day of stay**. Histogram represents the observed data ie the mean number of new prescribing errors per 10 order lines. The fitted curve with 95% confidence intervals represents the estimation of of the mean number of new prescribing errors per 10 order lines at *i*^*th *^day derived from the mixed Poisson regression model.

The histogram in Figure 1 shows the number of new prescribing errors per 10 order lines according to the day of stay. More than 51% of these errors (60/117) occurred on the day of admission (see figures below histogram). This rate was 80% over the first three days. Nine (7.7%) errors were observed in 8 of the 96 stays (8.3%) which lasted 8 days or more. These 9 errors were indifferently distributed between the 8^th ^and the 15^th ^day of stay (see Figure 1). The new prescribing errors rate per ten prescriptions order lines was maximum the day of admission and decreased in the first six days of stay. We observed an increase in the rate between the sixth and eighth day and after the fourteenth day but these fluctuations corresponded to small variations in the number of errors. The level of severity of errors was not significantly different in the first days of stay (71% were level C for the first 3 days and 84% for the next days).

The results from the GEE regression model are presented in Table 1. The backward selection process indicated that hypertension, thromboembolic disease, ward and day of discharge didn't influence the new prescribing errors rate in the stay. However, renal impairment, number of order lines and day of the stay were significantly associated with the risk of error. An error was twice likely to occur among patients with renal impairment than among patients without [OR = 2.2 (95% CI, 1.3-3.5), p = 0.002]. In addition, for a ten increase of the number of order lines in a prescription, the risk of error increased 3 times [OR = 3.1 (95% CI, 1.8-5.2), p < 0.001]. We found that the risk of a new prescribing error decreased significantly over time (p < 0.001). This means that the more distant the day was from the first day of stay, the less the risk was to have a new prescribing error. However, this decrease was not constant over time but "digressive" (modelled with a log-linearity relationship between the logit and the day). For example the reduction of the risk of a new prescribing error in the second day of stay compared to the first day was more important than the reduction of risk in the fifth day of stay compared to the fourth day. The mixed Poisson regression model of the new prescribing errors rate per order lines conducted to similar results. Figure 1 shows the estimation of the mean number of new prescribing errors per 10 order lines with 95% confidence intervals (CIs) derived from the final multivariate model (see Additional files 2 and 3 for the results from the univariate and multivariate analyses). There was a significant difference in the error rates between the 3 first days of stay (CIs disjoint). The renal impairment was significant in the model but the interaction term with the day of stay was not. This indicated that the mean number of new prescribing errors was different the day of admission for patients with and without renal impairment but the decrease was similar along the stay. Among patients with renal impairment, the mean number of new prescribing errors per 10 order lines was estimated to 0.62 (95% CI, 0.44-0.88) the day of admission, 0.21 (95% CI, 0.16-0.29) the second day of stay, 0.12 (95% CI, 0.08-0.17) the third day of stay, and less than 0.10 from the fourth day of stay. Among patients without renal impairment, the expected mean numbers were respectively 0.34 (95% CI, 0.24-0.48)], 0.12 (95% CI, 0.08-0.16)], and less than 0.07 from the third day of stay.

**Table 1:** Estimated Odds Ratio and 95% Confidence Intervals for the GEE* regression model for new prescribing error in a prescription.

GEE* regression model	Univariate analysis			Final multivariate model†		
**Variable**	**Crude Odds Ratio**	**[95% CI]**	**P value**	**Adjusted Odds Ratio**	**[95% CI]**	**P value**

**Number of order lines, ***for 10 lines increase*	2.34	[1.54-3.55]	<0.001	3.13	[1.89-5.16]	<0.001
**Day, ***for 1 day increase*	0.57	[0.47-0.70]	<0.001			
**Log(day)**‡			<0.001			<0.001
day2 *versus *day 1	0.39	[0.29-0.52]		0.34	[0.25-0.47]	
day3 *versus *day 2	0.57	[0.49-0.67]		0.54	[0.45-0.64]	
day4 *versus *day 3	0.67	[0.60-0.76]		0.64	[0.56-0.73]	
day5*versus *day 4	0.74	[0.67-0.81]		0.71	[0.64-0.79]	
day6 *versus *day 5	0.78	[0.72-0.84]		0.76	[0.69-0.82]	
day7 *versus *day 6	0.81	[0.76-0.86]		0.79	[0.73-0.85]	
**Renal failure**			<0.001			0.002
No	1.00			1.00		
Yes	2.19	[1.41-3.39]		2.16	[1.35-3.43]	
**Hypertension**			0.49			
No	1.00					
Yes	0.85	[0.54-1.34]				
**Thromboembolic disease**			0.061			
No	1.00					
Yes	1.65	[0.97-2.80]				
**Ward**			0.016			
diabetes care	1.00					
geriatrics	2.08	[0.62-6.99]				
internal medicine (ward 1)	2.48	[0.72-8.42]				
internal medicine (ward 2)	3.20	[0.95-10.65]				
immunology	3.33	[0.99-11.15]				
vascular medicine	3.41	[1.03-11.28]				
nephrology	6.31	[1.94-20.46]				
**Day of discharge**			0.124			
No	1.00					
Yes	0.49	[0.19-1.22]				

* GEE = Generalized Estimating Equations; † After backward selection with all potential confounders; ‡ The log-linearity relationship traduces that the decrease of the risk to have a new prescribing error was not constant over time but "digressive".

### Secondary outcome

**Figure 2:**
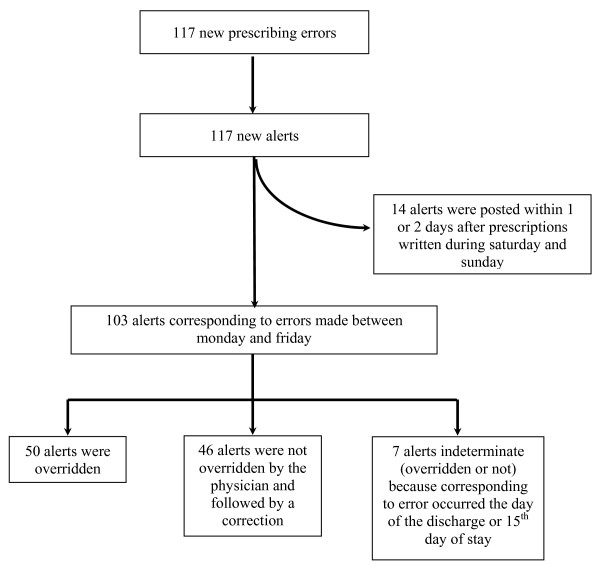
**Flow charts of the alerts**.

Pharmacists posted 117 alerts in response to the 117 new prescribing errors. Among these 117 alerts, 103 were posted the same day. The other 14 alerts were posted within 1 or 2 days after prescriptions written between Saturday and Sunday. These were excluded from further analyses (figure 2).

Characteristics of the 117 new prescribing errors and status of the alert the next day are described in Table 2. More than half of the alerts (56.4%) targeted inappropriate choice of drug and/or drug dose. Twenty four (20.5%) alerts targeted drug omissions. No alert highlighted life-threatening error.

**Table 2:** Description of the 117 new prescribing errors and status of the alert the next day.

Status of the alert the next day	Alert Overridden		Alert Not overridden		Indeterminate*		Alerts posted within 1 or 2 days after prescriptions †		All	
Error	N = 50		N = 46		N = 7		N = 14		N = 117	
**Type **-- no (%)										
Inappropriate choice of drug and/or drug dose	27	(55.6)	26	(57.4)	2	(28.6)	11	(78.6)	66	(56.4)
Drug-drug interaction	10	(20.0)	3	(6.5)	1	(14.3)	2	(14.3)	16	(13.7)
Wrong unit	4	(8.0)	1	(2.2)	1	(14.3)	1	(7.1)	7	(6.0)
Wrong route	1	(2.0)	1	(2.2)	0	(0.0)	0	(0.0)	2	(1.7)
Drug omission	8	(16.0)	14	(30.4)	2	(28.6)	0	(0.0)	24	(20.5)
Duplicate order	0	(0.0)	1	(2.2)	1	(14.3)	0	(0.0)	2	(1.7)
**Potential severity **-- no (%)										
Life-threatening	0	(0.0)	0	(0.0)	0	(0.0)	0	(0.0)	0	(0.0)
Significant or serious	14	(28.0)	7	(15.2)	2	(28.6)	8	(57.1)	31	(26.5)
None	36	(72.0)	39	(84.8)	5	(71.4)	6	(42.9)	86	(73.5)

* error occurring at the end of follow-up period (the day of discharge or 15th day of stay).

† alerts posted within 1 or 2 days after prescriptions written during the week end.

Among these 103 alerts, 7 occurred on the day of discharge or on the fifteenth day of the stay, so that it was not possible to evaluate whether or not they would have been overriden. Analysis of alert's overriding behavior therefore concerned the 96 remaining alerts (figure 2). Fifty (52%) alerts have been overridden (*i.e*. error remained uncorrected by prescriber the following day. Drug omissions were the most frequently alerts taken into account by prescribers: 64% (14/22) of these alerts were corrected.

**Figure 3:**
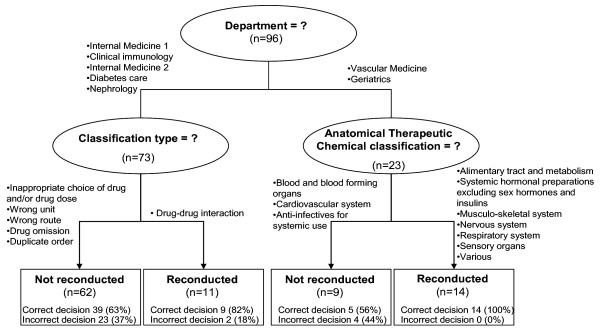
**CART tree for predicting alert's overriding among new alerts**. Numbers in ellipses and rectangles report the number of observed alerts. Classifying variables are indicated in the ellipses as questions and splitting rules are printed at the branches. In the terminal nodes (rectangles), the number and the proportions of correctly predicted outcomes by the tree (respectively incorrectly predicted) are presented as "correct decision" (respectively "incorrect decision"). The ward was the first discriminating variable, *i.e*. most influential reasons for alert's overriding. For the vascular medicine and geriatrics wards (23 alerts), alert's overriding was dependent from the first level of the 'Anatomical Therapeutic Chemical classification '. Among the alerts due to 'Alimentary tract and metabolism, Systemic hormonal preparations, excluding sex hormones and insulins, Musculo-skeletal system, Nervous system, Respiratory system, Sensory organs and Various' errors (n = 14), it correctly classified 100% of the alerts with an overriding on the next day. Among the errors belonging to the categories 'Blood and blood forming organs, Cardiovascular system, Anti-infectives for systemic use' (n = 9), it correctly classified 5 alerts (56%) with a non-overriding on the next day and missed 4 alerts' overriding. For the other wards (internal medicine 1, clinical immunology, internal medicine 2, diabetes care, and nephrology), the next differentiating factor was type of errors. For new alerts with 'Inappropriate choice of drug and/or drug dose, Wrong unit, Wrong route, Drug omission or Duplicate order' errors, the classification and regression tree predicted a non-overriding of the alert whereas for 'Drug-drug interaction' error, it anticipated an overriding on the next day.

Figure 3 shows the results of the classification and regression tree (CART) analysis. The final tree should be read from top to bottom. The 4 terminal nodes are presented as rectangles, whereas the nodes that are split further are presented as ellipses. The classifying variables selected by the model are indicated in the ellipses as questions and the splitting rules are printed at the branches that lead to the resulting nodes. Thus, each node represents a particular subset of alerts resulting from the application of all splitting rules that are higher in the tree structure. The numbers given within the nodes report the number of observed alerts in this node. In the terminal nodes, the number and the proportions of correctly predicted outcomes by the tree (respectively incorrectly predicted) are presented as "correct decision" (respectively "incorrect decision"). After removal of the new alerts which occurred on the day of discharge or on the fifteenth day of the stay, 96 alerts were classified among which 50 (52%) were alert's overriding the next day and 46 (48%) were not overridden. The ward was the first discriminating variable, *i.e*. most influential reasons for alert's overriding. For the vascular medicine and geriatrics wards (23 alerts), alert's overriding was dependent from the first level of the 'Anatomical Therapeutic Chemical classification'. Among the alerts due to 'Alimentary tract and metabolism, Systemic hormonal preparations, excluding sex hormones and insulins, Musculo-skeletal system, Nervous system, Respiratory system, Sensory organs and Various' errors (n = 14), it correctly classified 100% of the alerts with an overriding on the next day. Among the errors belonging to the categories 'Blood and blood forming organs, Cardiovascular system, Anti-infectives for systemic use' (n = 9), it correctly classified 5 alerts (56%) with a non-overriding on the next day and missed 4 alerts' overriding. For the other wards (internal medicine 1, clinical immunology, internal medicine 2, diabetes care, and nephrology), the next differentiating factor was type of errors. For new alerts with 'Inappropriate choice of drug and/or drug dose, Wrong unit, Wrong route, Drug omission or Duplicate order' errors, the classification and regression tree predicted a non-overriding of the alert whereas for 'Drug-drug interaction' error, it anticipated an overriding on the next day.

Two alerts which were not overridden were predicted as overridden by the tree (false positive) and 27 overridden alerts were predicted as not overridden (false-negative). This corresponds to a specificity of 96% (95% CI, 89-100), sensitivity of 46% (95% CI, 32-60), and a high positive likelihood ratio: 10.6 (95%CI, 2.6-42.4). Positive and negative predictive values were respectively 92% (95% CI, 81-100) and 62% (95% CI, 50-74).

## Discussion

The originality of this study lies in the analysis of the chronology of prescribing errors during hospital stay. We found that 0.9% of all drug orders during a period of 15 days were detected by pharmacists for potential prescribing errors. Among all errors, 25% were significant (level C) or serious (level B) and no one was life-threatening during the period of analysis. We found that 51% of them occurred the first day of stay and the error rate decreased drastically in the first three days of stay. Moreover, in our setting 80% of the errors occurred on the 3 first days. In practice, patients are admitted most often by junior doctors who try to know the medication history. This could explain the high proportion of errors that occured on the first day. In order to reduce this rate, we would like to perform a systematic pharmacist consultation with the patient the day of the admission in order to accurate medication history.

These results are in agreement with those reported by Cornish et *al*. et Bobb *et al*. studies who respectively found that 53% and 64% of prescribing errors occurred at the time of admission to the hospital [12,13]. Considering the risk of prescribing errors thereby shown to be maximal at admission and during the first three days of stay, the role of pharmacists in alerting for prescribing error could be focused during this period. This suggestion is strengthened by the fact that more than 80% occurring later during the stay have a level C of severity.

In a previous study performed in the same setting, we found a similar rate of prescribing errors which is consistent with various results published in other studies, whether they considered prescribing errors alerted by a CPOE [13,19] of by pharmacists [20,21]. Furthermore, renal impairment was significantly associated with the risk of error. Indeed, patients with renal impairment have a twice as high risk of error than patients without. These data were in agreement with other studies where drug dosage are inappropriate for 20 to 46% of prescriptions requiring dosage adjustments based on renal function [16,17].

The main limit of our study is the low number of new errors to model the evolution of the error incidence risk after the 5^th ^day, and this may alter the robustness of the chronological model. However, our data were collected prospectively on a daily basis through routine pharmacy validation tasks and from the list of alerts posted the same day.

We found that 52% of alerts were overridden. Two explanations of this important rate of overriden alerts could be discussed. First, clinical pharmacy is rapidly expanding in France and its impact among prescribers is still modest and little known. Additionally, as we observed in a previous study [11]), the alerts sent by the pharmacist are not readily visually accessible to the prescriber in our CPOE system. Five clicks are required to reach and read the content of alerts. Moreover, drug omissions were the alerts most frequently taken into account by prescribers. Classification and regression tree (CART), analysis showed that ward was the first discriminating variable influencing reasons for alert's overriding. Since all wards provide similar acute care in conventional hospital beds, this discriminating effect may be rather explained by different behaviors of medical staff and different degrees of collaboration with pharmacists. The ward, the Anatomical Therapeutic Chemical class of prescribed drug and the type of potential error allowed to predict the overriding of the alert with a positive predictive value of 92%. According to the classification and regression tree (CART) analysis and although the renal impairment is known as a risk factor of error, this criterion doesn't appear as predictive of the consideration of the posted alert.

The sensitivity of the classification and regression tree (CART) model was only of 46%. This analysis of predictive factors for alerts overriding is limited by the low number of new errors in our data. The qualification of alerts by type and severity was performed by five pharmacists well trained to routine validation tasks. The classification used was derived from published and commonly accepted ones [6,22-24] and used for several years by the pharmacists team [2,11].

Various studies have addressed the frequency and explanation of overriding alerts. In a systematic review of 17 studies, Van der Sijs *et al*. identified 9 studies which quantitatively analysed overriding in hospital setting [25]. The reported overriding rate varied from 40% to 96% and the alerts most often overridden concerned orders renewals, interaction with topical drugs, and uncertain allergies. All these studies concentrated on alerts automatically targeted by a CPOE. In another qualitative study, Van der Sijs investigated which types and objects of alerts were deemed useless enough by clinicians to be turn off in the CPOE system. For three among the 24 alerts studied, more than 50% of clinicians agreed to turn them off: 1) coumarins and amiodarone or propafenone, 2) beta-blockers and non-steroidal anti-inflammatory drugs and 3) selective beta-blockers and insulin [26].

## Conclusions

In the French regulation context of drug prescription and pharmacy validation, this study can help the pharmacists to orient their time and resource investments on validation tasks. Since 80% of the prescribing errors occurring on the first 3 days of the hospital stay, the analysis of the drug computerized orders by pharmacists should concentrate over this period. The restriction of the analysis of prescriptions to the first day of stay may be safer than the daily analysis all along the patient stay.

Moreover, the implementation of alerts to the physician could complement the pharmacist's validation tasks and make the prevention of prescribing errors more efficient. In this perspective, the difference of overriding behavior between wards and according drug Anatomical Therapeutic Chemical class or type of error should guide the choice and specification of these alerts.

## Competing interests

The authors declare that they have no competing interests.

## Authors' contributions

TC extracted and analysed the data and prepared a draft of the manuscript and contributed to all other aspects of the study. IC prepared a revised version of the manuscript and provided intellectual input into study design and statistical analysis. FG performed the statistical analyses. VB extracted the data. DB participated in the design of the study. PP and PD contributed to the final version of the manuscript. BS participated in the design of the study, the critical revision of the manuscript and its supervision. All authors have given final approval of the submitted manuscript.

## Pre-publication history

The pre-publication history for this paper can be accessed here:

http://www.biomedcentral.com/1472-6963/10/13/prepub

## Supplementary Material

Additional file 1 **Description of the classifications for type and severity of prescribing errors**. This file is a classification of the type and degree of severity of the prescribing errors. Click here for file

Additional file 2 **Estimated mean number of new prescribing errors per 10 order lines and 95% Confidence Intervals for the univariate Poisson regression model**. Click here for file

Additional file 3 **Estimated mean number of new prescribing errors per 10 order lines and 95% Confidence Intervals for the multivariate Poisson regression model**. Click here for file
