# Supplementary material for: Chronology of prescribing error during the hospital stay and prediction of pharmacist's alerts overriding: a prospective analysis
Source: BMC Health Serv Res. 2010 Jan 12;10:13. doi: 10.1186/1472-6963-10-13 (PMC2820036; doi:10.1186/1472-6963-10-13)
Supplement: Additional file 1 — Description of the classifications for type and severity of prescribing errors. This file is a classification of the type and degree of severity of the prescribing errors. [file 1472-6963-10-13-S1.DOC]

Appendix 1 : Description of the classifications for type and severity of prescribing errors.

| Classification | Advice or correction about: | Example |
| --- | --- | --- |
| **Type** |  |  |
| Inappropriate choice of drug and/or drug dose (InC) | - Non adaptation to biological or pharmacological follow-up according to official recommendations or specific protocols  - A patient’s characteristic known to be a contra-indication to the treatment  - An improper or missing dose by day or by each drug intake | - Treatment by Acenocoumarol non adapted to patient INR  - Treatment by amoxicillin, whereas the patient is allergic |
| Drug-drug interaction (Int) | A potential interaction with current drug regimen | Risk of ergotism induced by interaction of erythromycin with dihydroergotamine. |
| Reconstitution (Rec) | An improper or missing indication for reconstitution or injection (bolus, infusion, …) | “Amikacin 500mg.” Mentioned alone or associated with chloride sodium solution, whereas the prescription needed is “dilution in 200mL of dextrose 5%, infusion time: 30 minutes.” |
| Wrong unit (WU) | An improper unit of administration | Warfarin at 5mg per os : 10 *pill* at 6:00 pm;.instead of 10*mg* |
| Wrong route (WR) | An improper route of administration, according to official recommendations or specific protocols | Subcutaneous bolus of amikacin whereas some reports of cutaneous necrosis are reported in litterature. |
| Drug omission (DOm) | -A drug omitted by the prescriber | Sevelamer omitted in a patient with chronic kidney disease and hyperphosphatemia |
| Duplicate order (DOr) | Duplication of a drug order | Levothyroxin prescribed twice for the same patient |
|  | | |
| **Potential severity** |  |  |
| Life-threatening (A) |  | Warfarin at 5mg per os : 10 *pill* at 6:00 pm;.instead of 10*mg* |
| Significant or serious (B) |  | Only written: “Amikacin 500mg.”  Need to add: “dilution in 200mL of dextrose 5%, infusion time: 30 minutes.” |
| None (C) | systematic alert on usage precaution in absence of explicit contra indication | Creatinine clearance is 397micromol/L, adverse events of morphine have to be monitored closely |
