# Supplementary material for: Chronology of prescribing error during the hospital stay and prediction of pharmacist's alerts overriding: a prospective analysis
Source: BMC Health Serv Res. 2010 Jan 12;10:13. doi: 10.1186/1472-6963-10-13 (PMC2820036; doi:10.1186/1472-6963-10-13)
Supplement: Additional file 2 — Estimated mean number of new prescribing errors per 10 order lines and 95% Confidence Intervals for the univariate Poisson regression model. [file 1472-6963-10-13-S2.DOC]

Appendix 2 : Estimated mean number of new prescribing errors per 10 order lines and 95% Confidence Intervals for the univariate Poisson regression model.

| **Poisson regression model** | **Univariate analysis** | | |
| --- | --- | --- | --- |
| **Variable** | **Estimated mean number of new prescribing errors per 10 order lines*** | **[95% CI]** | **P value** |
| **Day**† |  |  | <0.001 |
| day1 | 0.367 | [0.276-0.488] |  |
| day2 | 0.193 | [0.157-0.237] |  |
| day3 | 0.102 | [0.075-0.137] |  |
| day4 | 0.053 | [0.033-0.085] |  |
| day5 | 0.028 | [0.015-0.054] |  |
| day6 | 0.015 | [0.006-0.035] |  |
| day7 | 0.008 | [0.003-0.022] |  |
| **Log(day)** ‡ |  |  | <0.001 |
| day1 | 0.415 | [0.314-0.549] |  |
| day2 | 0.145 | [0.116-0.180] |  |
| day3 | 0.078 | [0.057-0.106] |  |
| day4 | 0.051 | [0.034-0.075] |  |
| day5 | 0.036 | [0.022-0.058] |  |
| day6 | 0.027 | [0.016-0.047] |  |
| day7 | 0.022 | [0.012-0.039] |  |
| **Renal failure** |  |  | 0.008 |
| No | 0.180 | [0.138-0.235] |  |
| Yes | 0.106 | [0.080-0.141] |  |
| **Hypertension** |  |  | 0.161 |
| No | 0.109 | [0.079-0.151] |  |
| Yes | 0.147 | [0.113-0.191] |  |
| **Thromboembolic disease** |  |  | 0.40 |
| No | 0.149 | [0.105-0.209] |  |
| Yes | 0.124 | [0.097-0.157] |  |
| **Ward** |  |  | 0.54 |
| diabetes care | 0.089 | [0.024-0.324] |  |
| geriatrics | 0.104 | [0.060-0.181] |  |
| internal medicine (ward 1) | 0.138 | [0.078-0.244] |  |
| internal medicine (ward 2) | 0.130 | [0.082-0.206] |  |
| immunology | 0.105 | [0.065-0.169] |  |
| vascular medicine | 0.135 | [0.087-0.210] |  |
| nephrology | 0.188 | [0.127-0.279] |  |
| **Day of discharge** |  |  | 0.176 |
| No | 0.072 | [0.030-0.173] |  |
| Yes | 0.135 | [0.109-0.166] |  |

* We estimated the mean number of new prescribing errors per 10 order lines since the median number of order lines in a prescription was 7.

† The mean number of new prescribing errors was reduced by 47% each day

‡ The log-linearity relationship traduces that the decrease of the mean number of new prescribing errors was not constant over time but “digressive” (65% between the first and the second days, 46% between the second and third days...).
