# Supplementary material for: Chronology of prescribing error during the hospital stay and prediction of pharmacist's alerts overriding: a prospective analysis
Source: BMC Health Serv Res. 2010 Jan 12;10:13. doi: 10.1186/1472-6963-10-13 (PMC2820036; doi:10.1186/1472-6963-10-13)
Supplement: Additional file 3 — Estimated mean number of new prescribing errors per 10 order lines and 95% Confidence Intervals for the multivariate Poisson regression model. [file 1472-6963-10-13-S3.DOC]

Appendix 3 : Estimated mean number of new prescribing errors per 10 order lines and 95% Confidence Intervals for the multivariate Poisson regression model.

| **Final multivariate Poisson regression model**† | **Estimated number of**  **new prescriber errors per 10 order lines*** | **95% CI** |
| --- | --- | --- |
| *All patients* |  |  |
| Day1 | 0.396 | [0.296-0.530] |
| Day2 | 0.136 | [0.108-0.172] |
| Day3 | 0.073 | [0.053-0.100] |
| Day4 | 0.048 | [0.032-0.071] |
| Day5 | 0.033 | [0.021-0.054] |
| Day6 | 0.025 | [0.015-0.043] |
| Day7 | 0.020 | [0.011-0.037] |
| *Patients with renal failure*‡ |  |  |
| Day1 | 0.622 | [0.444-0.873] |
| Day2 | 0.215 | [0.162-0.284] |
| Day3 | 0.115 | [0.081-0.163] |
| Day4 | 0.074 | [0.048-0.113] |
| Day5 | 0.053 | [0.032-0.086] |
| Day6 | 0.040 | [0.023-0.069] |
| Day7 | 0.031 | [0.017-0.057] |
| *Patients without renal failure*‡ |  |  |
| Day1 | 0.337 | [0.240-0.473] |
| Day2 | 0.116 | [0.087-0.156] |
| Day3 | 0.062 | [0.043-0.090] |
| Day4 | 0.040 | [0.026-0.062] |
| Day5 | 0.028 | [0.017-0.047] |
| Day6 | 0.022 | [0.012-0.038] |
| Day7 | 0.017 | [0.009-0.032] |

* We estimated the mean number of new prescribing errors per 10 order lines since the median number of order lines in a prescription was 7.

† After backward selection with all potential confounders, the significant variables were the day (with a log-transformation) and the renal failure.

‡ The renal impairment was significant in the model but not the interaction term with the day of stay. This indicated that the mean number of new prescribing errors was different the day of admission for patients with and without renal impairment but the decrease was similar along the stay.
